# Can't play, won't play: longitudinal changes in perceived barriers to participation in sports clubs across the child–adolescent transition

**DOI:** 10.1136/bmjsem-2015-000079

**Published:** 2016-03-21

**Authors:** Laura Basterfield, Lauren Gardner, Jessica K Reilly, Mark S Pearce, Kathryn N Parkinson, Ashley J Adamson, John J Reilly, Stewart A Vella

**Affiliations:** 1 Institute of Health & Society and Human Nutrition Research Centre, Newcastle University, Newcastle upon Tyne, UK; 2 Early Start Research Institute, Faculty of Social Sciences, University of Wollongong, Wollongong, New South Wales, Australia; 3 Institute of Health & Society, Newcastle University, Newcastle upon Tyne, UK; 4 Physical Activity for Health Group, University of Strathclyde, Glasgow, UK

**Keywords:** Sports, Children's health and exercise, Adolescent, Epidemiology

## Abstract

**Background:**

Participation in sports is associated with numerous physical and psychosocial health benefits, however, participation declines with age, and knowledge of perceived barriers to participation in children is lacking. This longitudinal study of children and adolescents aimed to use the ecological model of physical activity to assess changes in barriers to participation in sports clubs to identify age-specific and weight-specific targets for intervention.

**Methods:**

Longitudinal study—Perceived barriers to sports participation were collected from a birth cohort, the Gateshead Millennium Study (n>500) at ages 9 and 12 years. The open-ended question ‘Do you find it hard to take part in sports clubs for any reason?’ was completed with free text and analysed using content analysis, and the social–ecological model of physical activity.

**Results:**

Barriers from across the social-ecological model were reported. Barriers at 9 years were predominantly of a physical environmental nature, and required high parental involvement (for transport, money, permission), or were associated with a lack of suitable clubs. At 12 years, perceived barriers were predominantly classed as intrapersonal (‘they're boring’) or social environmental (‘my friends don't go’). Perceived barriers were not associated with weight status.

**Conclusions:**

Perceived barriers to sports participation change rapidly in childhood and adolescence. Future interventions aiming to increase sports participation in children and adolescents should target specific age groups, should consider the rapid changes which occur in adolescence, and aim to address prominent barriers from across the socioecological model. Perceived barriers may be unrelated to current weight status, allowing for more inclusive solutions.

**What are the new findings**

- Children's perceived barriers to sport participation change over a short space of time.
- Perceived barriers may be unrelated to weight status.
- Perceived barriers may be similar for boys and girls before adolescence.

## Introduction

Physical activity has been associated with numerous health benefits during childhood and adolescence.^1^ Sport is one of the most popular forms of physical activity worldwide,^2^ it contributes 55–65% of daily moderate to vigorous energy expenditure in youth,^3^ ^4^ and conveys a range of psychosocial health benefits that are over and above those attributable to physical activity.^5^ ^6^ However, as 20–30% of children in the UK, the USA and Australia do not participate in sport,^7–9^ and participation rates decline dramatically as children age, it is essential to investigate the barriers preventing participation in youth sport.^10^ ^11^ Much of the current research focuses on reasons for participation and dropout.^11–13^ However, the understanding of barriers preventing participation from the child or adolescent's perspective is important. The ecological model of health behaviour was developed to ‘emphasize the environmental and policy concepts of behaviour, while incorporating social and psychological influences’.^14^ The social–ecological model of physical activity (EMPA) was developed from this, and ‘portrays physical activity behaviour as being influenced by interplay between environmental settings and biological and psychological factors’,^15^ providing a framework for designing and evaluating physical activity and other health-related behavioural interventions. The model comprises four main domains: intrapersonal (individual beliefs, knowledge, skills, age), social environment (relationships, culture, society), physical environmental (natural or man-made environments) and policy (legislation). Barriers to sports participation may fall into any of these domains. A review of the relatively small amount of research in the area found that adolescents perceived social and intrapersonal factors to be the most prominent barriers to sports participation.^16^ However, these studies involved cross-sectional research designs, and there is no research into how perceived barriers to participation change over time in the same children, and data on children's barriers (rather than adolescents’) is lacking.^16^ Barriers would be expected to change as children develop both physically and socially; what they gain in motor skills and sports knowledge^17^ may be tempered by a new awareness of social standing and peer influence.^18^

In our recent prospective study in an English birth cohort,^19^ continued participation in sports clubs between the ages of 9 and 12 years was associated with decreased adiposity. This finding is particularly important given the high prevalence of obesity and associated negative outcomes among youth.^9^ Additionally, this finding suggests that individuals with increased adiposity may be less likely to participate in sports, and body-related or social barriers may be particularly prominent in this group.^20^ Similar barriers may be more evident among females,^21^ as girls and young women are less physically active and take part in less sport than their male peers.^22^ ^23^

The novel aim of this study was to investigate how perceived barriers to participation in school and outside-school sports clubs change in the same cohort over 3 years. This information would allow interventions to be tailored to the specific needs of children and adolescents at a time of high risk of dropout. Three main hypotheses were tested: (1) perceived barriers will change from 9 to 12 years; (2) overweight children will perceive different barriers to children of healthy weight and (3) girls will perceive different barriers than boys.

## Methods

### Sample

The Gateshead Millennium Study is a birth cohort of adolescents born in northeast England between June 1999 and May 2000.^24^ Briefly, all children born to Gateshead-resident mothers in 34 prespecified recruiting weeks were invited to participate with no exclusion criteria. In total, 1029 babies were recruited to the original cohort, 523 male (50.8%) 506 female (49.2%) predominantly from the white ethnic majority (98%). The metropolitan borough of Gateshead is a mix of urban and rural, and the northeast of England is more deprived than England in general.^25^ The sample has remained socioeconomically representative of northern England and stable throughout the study; at birth, 15% of the sample was in the most affluent quintile, 20% in the second most affluent quintile, then 23%, 22% and finally 19% of the sample in the least affluent quintile. At follow-up at age 9 years, 18% of the sample were in the most affluent quintile, followed by 22%, 22%, 20% and then 18% in the least affluent quintile.^24^ The northeast consistently has higher than national average levels of childhood obesity.^26^

Data for the current analyses were collected at two datasweeps: at ages 8–10 and 11–13 years, referred to as 9 and 12 years.^19^ For each phase, families who had not previously opted out of the cohort were sent an invitation letter and information leaflet. Informed consent was obtained from the main carer of each child; children provided written assent. Ethical approval for the study was granted by the Newcastle University Ethics Committee.

### Measures: barriers to sport participation

Children completed the Youth Sport Survey (adapted from Godin and Shepherd 1985^27^), about sports clubs they attended both in school and outside school.^19^ For this study, 72% of children at 9 years (n=421), and 63% of children at 12 years (n=331) took part in a sports club, and participation at 12 years was associated with reduced fat mass index.^19^ The children also answered the question ‘Do you find it hard to take part in sports clubs for any reason?’ with free text answers for both school sports clubs and outside-school sports clubs. The responses were analysed using content analysis using an inductive thematic approach: the answer was read and the ‘theme’ or ‘subdomain’ associated with it was created. For example, the answer ‘I don't take part in any outside school clubs, because none of my friends do’, was used to create a subdomain of ‘friends don't go’, other similar responses were then placed into this category. Subdomains were then grouped thematically according to a simplified version of EMPA^15^ that consisted of three main domains: physical environmental, intrapersonal and social environment. A cumulative EMPA count was created for each domain of EMPA. A response could be placed into more than one category of EMPA if it included more than one factor. Subdomains were not created a priori, as we did not know what answers would be given.

Data were coded independently by the lead author and a research assistant, who then met to discuss and agree on any discrepancies and those that were difficult to code.

### Measures: anthropometry

At each time point, height was measured to 0.1 cm with a Leicester height measure (Chasmors, London, UK), and weight to 0.1 kg in light indoor clothing. Body mass index (BMI) centiles and BMI z-scores according to age-specific UK 1990 data^28^ were derived, and children categorised into healthy weight (HW, <85th centile), overweight (OW, ≥85th <95th centile) or obese (OB, ≥95th centile). Stage of puberty was assessed at 12 years using the self-reported Pubertal Development Scale,^29^ a self-report measure of puberty for young adolescents with good reliability and validity.

### Statistical analyses

Data were analysed in SPSS V.21 and STATA V.13. Answers were analysed by EMPA domain and overweight status. To assess the change in reported barrier over time, a change variable was created; children either changed domain of EMPA or did not, and within-subjects longitudinal associations were then examined using logistic regression. χ^2^ Tests were used to assess the association of EMPA domain with overweight status. The influence of puberty at 12 years, and socioeconomic status (SES) (measured by Townsend score, an area-based measure derived from residential postcode,^30^ and divided into quintiles) was tested with one-way ANOVA. Differences between sexes were tested by logistic regression.

## Results

At 9 years of age, 574 children took part, and at age 12 years, 500 adolescents took part, and 441 children answered the questions at both 9 and 12 years. There were no differences in BMI or BMIz-score between children who took part at both time points and those who did not, but results are restricted to those with data at both 9 and 12 years. Participant characteristics are shown in table 1.

**Table 1:** Participant characteristics, n=441

	9 Years		12 Years	
Male (n, %)	210	47.6	210	47.6
Female (n, %)	231	52.4	231	52.4
Body mass index (BMI) (mean, SD)	17.9	2.8	20.5	3.8
BMI z-score* (mean, SD)	0.5	1.1	0.7	1.2
Healthy weight (n, %)	297	67.7	274	62.7
Overweight† (n, %)	80	18.2	82	18.8
Obese† (n, %)	62	14.1	81	18.5
Stage of puberty‡	–	–	2.3	0.6

*z-Scores calculated relative to age-specific UK 1990 reference data.^28^

†Cut-points for population monitoring of overweight were used to categorise the children into healthy weight (<85th centile), overweight (≥85th <95th centile) or obese (≥95th centile). Overweight and obese categories were combined for this analysis.

‡Continuous scale from 1 to 4; prepubertal to postpubertal.

**Figure 1:**
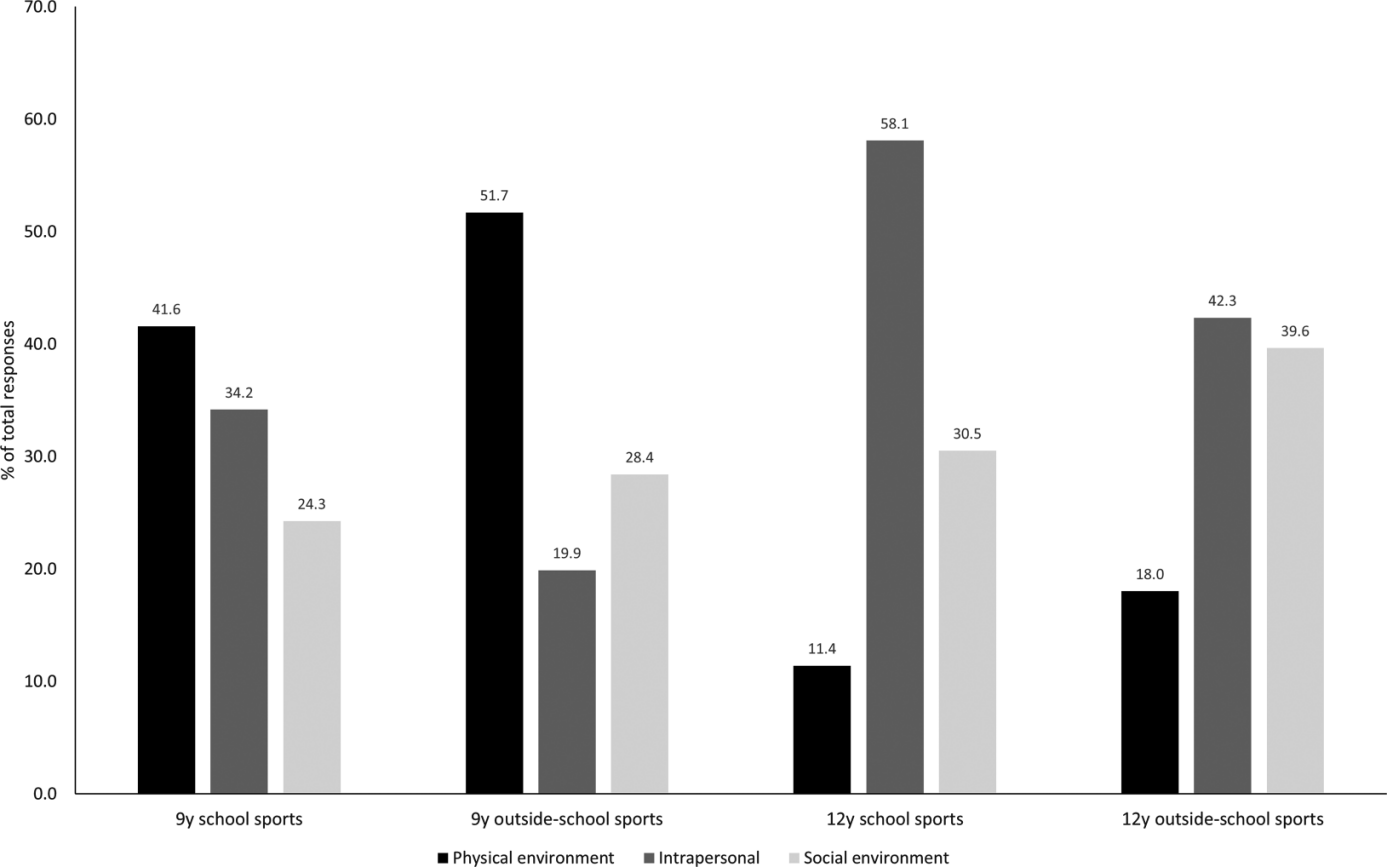
Distribution of responses by domain of social–ecological model of physical activity. Data are per cent of total responses for each domain of social–ecological model of physical activity, for each category of school and outside-school sports clubs, at each age (‘no reported barrier’ has been removed for clarity).

Responses to the question ‘Do you find it hard to take part in sports clubs for any reason?’ are described by socioecological domain of EMPA (figure 1). Children who reported no perceived barriers represented a substantial portion of the responses (40–60%), and were included in the analysis to reduce bias.

The number of subdomains within each domain of EMPA differed by age and by school or outside-school sports clubs; 46 subdomains were identified in total, there were 24, 24, 29 and 28 subdomains for 9 years school, 9 years outside-school, 12 years school, and 12 years outside-school sports clubs, respectively (see online supplementary table S2).

10.1136/bmjsem-2015-000079.supp1 Supplementary table 2 Reported barriers to participation in school- and outside-school sports clubs, by domain and sub-domain of the social-ecological model of physical activity*

Online supplementary table S2 shows how perceived barriers to taking part in sports clubs changed over 3 years. For younger children, the physical environmental domain was prominent, suggesting more practical difficulties, such as a lack of suitable club, for example, ‘[I would like to play] Tennis—isn't a club’ (ID1, boy, 9 years), ‘[I did] Street dance-not many people took part so it stopped’ (ID2, girl, 9 years), a lack of permission or transport: ‘[I would like to do] karate & football—[but there is] no one to take me’ (ID3, boy, 9 years), but also that clubs were only available to older or younger children. Lack of time was an issue at both ages: ‘I do nothing out of school or after school—not enough time’ (ID5, girl, 12 years). By adolescence, the respondents showed a marked change in their responses, with answers predominantly intrapersonal and socially environmental, and displaying a general lack of interest: ‘because … I can't be bothered to stay after school’ (ID4, boy 12 years), and having other interests and priorities, whether friends, family or homework: ‘I always have a lot of things to do at break and lunch, and none of the after school clubs suit me’ (ID6, girl, 12 years).

At both ages, there were children and adolescents describing themselves as ‘not sporty’, or disliking sports, and at 12 years worrying about not fitting in: ‘Sometimes I feel as if I won't fit in with others or maybe feel left out’ (ID7, boy, 12 years), ‘I don't feel comfortable and don't like doing things without my friends’ (ID8, girl, 12 years), ‘I am often very shy and rarely speak for myself. I am worried that I will get things mixed up so try to avoid things’ (ID9, girl, 12 years). There were reports about lack of fitness: ‘My chest really hurts when I exercise for too long’ (ID10, girl, 12 years), and also some starkly honest responses, for example, ‘my mum won't pick me up because she doesn't care’ (ID11, girl, 12 years), and: ‘because the internet exists’ (ID12, girl, 12 years).

To assess within-subject change over time, only the first answer given was used, as the majority of children gave only one answer (15 and 12 children at 9 years gave a second answer for school and outside-school sports clubs, respectively, and 13 and 8 children at 12 years). The within-subjects change over time found that 154 of 435 children identified barriers within the same domain of EMPA for school sports clubs from 9 to 12 years, and 160 of 420 for outside-school sports club (table 2). The distributions were also different between school and outside-school sports clubs at both ages, although the changes in EMPA domain from 9 to 12 years were not statistically significant (χ^2^ p=0.054 for outside-school sports clubs and p=0.410 for school sports clubs).

**Table 2:** Change in reported barrier to (a) school sports club participation and (b) outside-school sports club participation from age 9 to 12 years, by social-ecological model of physical activity (EMPA) domain

9 years EMPA domain	Physical environment	Intrapersonal	Social environment	No barrier reported	Total
*(a) 12 years EMPA domain*					
Physical environment	7	25	12	32	76
Intrapersonal	6	26	6	25	63
Social environment	3	16	13	16	48
No barrier reported	13	82	48	105	248
Total	29	142	86	178	435
*(b)*					
Physical environment	13	18	11	41	83
Intrapersonal	2	12	4	16	34
Social environment	5	8	8	26	47
No barrier reported	15	51	60	130	256
Total	35	83	89	213	420

**Figure 2:**
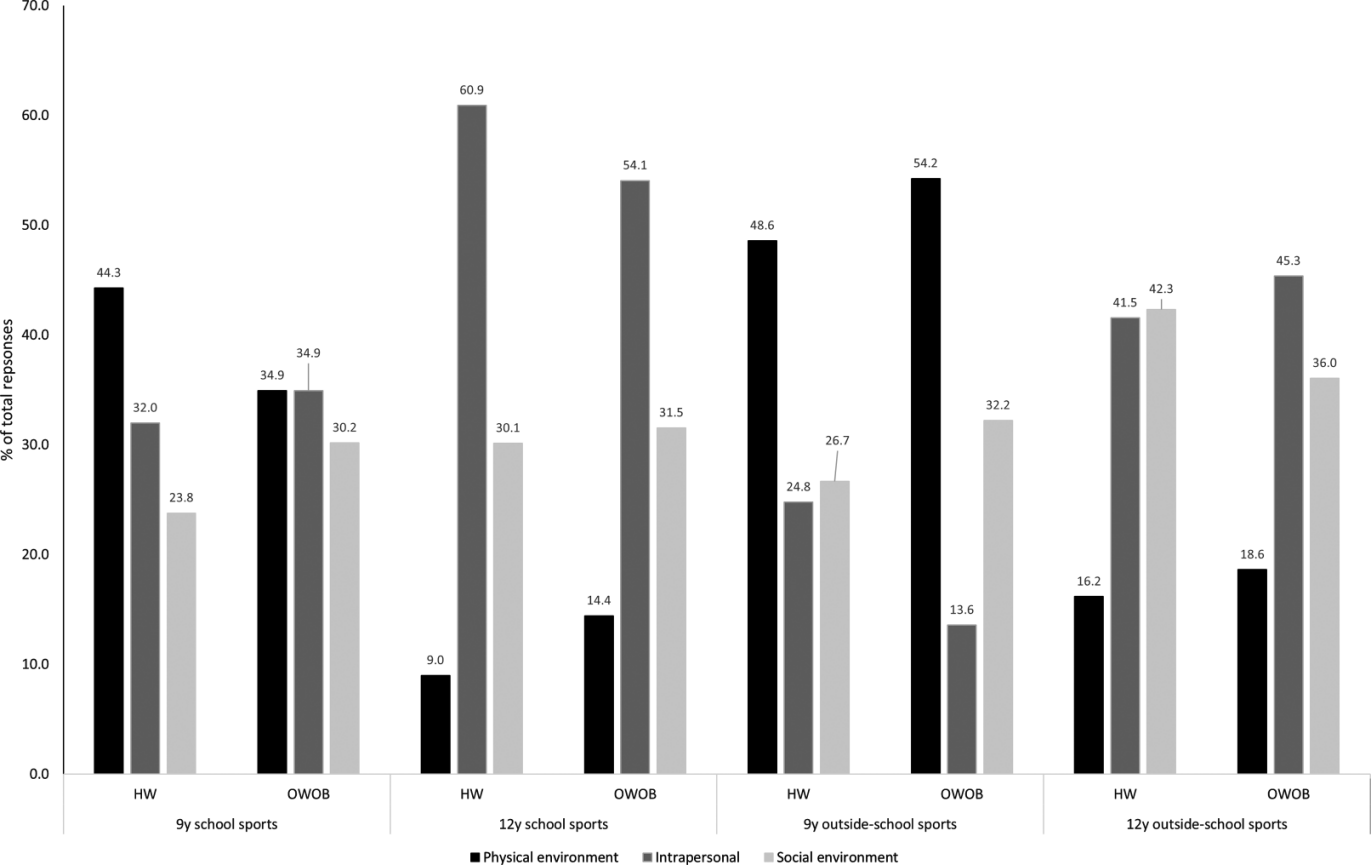
Distribution of responses by domain of social–ecological model of physical activity and overweight status. Data are per cent of total responses for each domain of social–ecological model of physical activity, for each category of school and outside-school sports clubs, at each age, by weight status (‘no reported barrier’ has been removed for clarity). HW, healthy weight; OWOB, overweight or obese.

Analysis by overweight status is shown in figure 2. There were no statistically significant differences between HW and OWOB children at each time point, however, change in perceived barrier was associated with OWOB status at 12 years, after controlling for sex, puberty and baseline OWOB status (OR 1.7, 95% CI to 1.0–3.0, p=0.033). This was due to more OWOB children reporting ‘no perceived barrier’ at 9 years, then a barrier at 12 years, than HW children (26% vs 34%).

The distribution of answers by EMPA was similar between the sexes, with the exception of outside-school sports clubs at 9 years, where girls gave fewest responses in the intrapersonal domain, but boys’ responses were split equally between intrapersonal and social environment. No statistically significant differences between the sexes were found either cross-sectionally at each age, or in the change in EMPA exhibited longitudinally when tested by χ^2^ (p>0.05), or by stage of puberty at 12 years (one-way ANOVA p>0.05). There were no associations with SES.

## Discussion

The current study is novel in using responses from the same group of children, at 9 years and then 12 years of age, and highlights the range of perceived barriers—46 were identified across just a 3-year timespan. The current work has also addressed an important gap in the literature by contributing to the knowledge of younger children's experiences.

### Age-related changes

There was a clear difference with age; as children, the perceived barriers were predominantly of a physical environmental nature. This is in keeping with children of this age and stage of development; they are not yet overly concerned about their social standing,^18^ and parental support is vital, as the children may be considered too young to travel home alone, do not have their own money (or parents cannot afford clubs/equipment), may forget to get permission forms completed, or are refused permission: ‘[I would like to go] swimming—Mum won't let me go back’ (ID 13, boy, 9 years). Others have found that parental support, plus access to a variety of clubs, are motivators for young children's participation in sports.^16^ A lack of time was cited at both ages, and at 9 years, this may have been due to homework or other clubs they enjoyed: ‘[I would like to do] karate—but clashes with piano lessons’ (ID 14, girl, 9 years). Lack of time and competing demands were reported by Canadian parents of children this age,^31^ and children in Ireland who had never participated in sports clubs provided similar reasons; they struggled to find suitable clubs, with transport, and with feelings of incompetence.^32^ Participants in the current study also reported that they ‘weren't good at sport’, or that it was ‘too hard’, and there were children with injuries, or who were scared of getting hurt. This could point to exposure to developmentally inappropriate sports,^17^ or that the children lacked the fundamental movement skills required to perform them. These could be difficult feelings to overcome, perhaps helped by improved teacher and coach training in understanding and assessing fundamental movement skills,^33^ and communicating their importance to parents.

The transition from primary to secondary school (and from childhood to adolescence), marked a clear distinction in barriers, with the most prominent subdomains capturing disinterest. Other researchers have emphasised the need for ‘sampling’ at these ages, where children try different sports with an emphasis on fun and participation, rather than competition.^34^ In the current study, many children already participated in a sports club, perhaps explaining why many children did not perceive any barriers. However, several responses could be intervention targets, including providing transport home, making clubs free/cheaper, and making clubs available to children of all ages. The results of this study also suggest that interventions to increase sports participation should be age-specific. The 12-year-olds’ concerns relating to their social environment emphasise the importance of friendship groups at this age: ‘I don't take part in any outside school clubs, because none of my friends do’ (ID15, girl, 12 years). This emerges during early adolescence, as they become more aware of what their friends think of them, and the need to feel accepted and similar.^18^ This has been described in a dance intervention for girls^35^; the authors suggest emphasising enjoyment and socialisation in recruitment campaigns.^35^ Peer acceptance and friendship quality are two important dimensions of peer influence that have been linked with increased commitment to sports, greater enjoyment, and improved psychosocial well-being among adolescents.^36^ ^37^

Some of the findings of this study echo those of Stanley *et al*^38^ ^39^ who discussed physical activity (not specifically sport participation) with children aged 10–13 years in Australia. The children mentioned the importance of friends, parental support, lack of time and perceived enjoyment,^38^ ^39^ highlighting a degree of generalisability of studies to this age group, enabling successful sports interventions developed in one setting to be applied more rapidly in others.

### Weight-related and sex-related barriers

The analysis found no clear difference with overweight status, suggesting that children and young adolescents have similar concerns across the weight spectrum. This may help future intervention designs to be more inclusive and is consistent with the finding that there is no difference in participation in organised sports by weight status.^40^ Similarly, sport participation has previously been shown to differ by SES,^19^ while other research has found that SES and cultural background, but not BMI, predict dropout from sport.^41^ There was also no clear association with sex, which is surprising, given that levels of physical activity and sports participation are lower in girls than boys. Allender *et al*^16^ reviewed barriers reported by young women, however, few of them were replicated in the current study. This may reflect the younger age of the current participants, and that sports clubs at 9 years may be available to both sexes. Further follow-up will show how perceived barriers change again.

### Strengths and limitations

Strengths include the relatively large sample size, with repeated assessment over 3 years, across the key period of transitions from primary–secondary school and childhood–adolescence. The use of a simple tool to extract information could be perceived as a weakness, but the breadth of answers, and similarity with qualitative studies shows that a simple question could help to inform intervention design. Other limitations include a lack of representation from across the entire socioecological model; information on policy barriers was limited, but might have included ‘lack of clubs’ or ‘expense’, however, more information would be required to decide that. An additional qualitative study with alternative methods, for example, interviews, might have revealed insights that were not possible from the current method. Additional information across the entire range of adolescence would also add value, therefore, it would be useful to follow the cohort further into adolescence and adulthood. Generalisability to other settings may be affected by the predominantly white ethnic background of the participants, and the low SES of the region; more affluent families or regions may have more clubs available to them.

### Setting this research in context

The importance of sports participation throughout life is being recognised by individual governments and also internationally, by the WHO Europe network for health-enhancing physical activity (HEPA), The Association For International Sport for All (TAFISA) and the IOC. The recent report by the UK Government ‘Sporting Future: A New Strategy for an Active Nation’^42^ promises a commitment to non-elite elite sport, and to increasing the funding and coaching available to children from 5 years old, as well as encouraging sport for social and mental health benefits. The current study should feed into the knowledge base for those seeking to increase sports participation in children and adolescents, specifically by understanding the variety of barriers and pressures that children and young people face.

## Conclusions

The transition from childhood to adolescence represents a marked change in perceived barriers to participation in sports clubs that may not differ substantially by sex or weight status. Interventions need to be tailored to the specific needs of the age group that is being targeted, and cover as many domains of the socioecological model as possible. Furthermore, interventions should address a narrow range of ages, because a universal intervention is unlikely to be applicable to both 9-year-olds and 12-year-olds. The views of children and adolescents should be sought prior to, and during, intervention design and implementation.
