# Supplementary material for: Can't play, won't play: longitudinal changes in perceived barriers to participation in sports clubs across the child–adolescent transition
Source: BMJ Open Sport Exerc Med. 2016 Mar 21;2(1):e000079. doi: 10.1136/bmjsem-2015-000079 (PMC5117043; doi:10.1136/bmjsem-2015-000079)
Supplement: Supplementary table 2 — Reported barriers to participation in school- and outside-school sports clubs, by domain and sub-domain of the social-ecological model of physical activity* [file bmjsem-2015-000079supp_table2.pdf]

Table 2. Reported barriers to participation in school- and outside-school sports clubs, by domain and sub-domain of the social-ecological model of physical activity*

Main domain of Social-Ecological Model of Physical Activity	Sub-domain	9y school sports clubs		9y outside-school sports clubs		12y school sports clubs		12y outside-school sports clubs	
		n	(%)	n	(%)	n	(%)	n	(%)
**Physical environment**	Distance	3	1.5	5	2.8	5	1.8	-	-
	Expense	5	2.5	12	6.8	-	-	8	3.6
	Need permission/forgot forms	13	6.4	19	10.8	-	-	1	0.5
	Club stopped/seasonal/weather	23	11.4	8	4.5	4	1.5	-	-
	No club/no equipment	29	14.4	15	8.5	3	1.1	8	3.6
	Facilities closed or unavailable	-	-	6	3.4	-	-	-	-
	Not sure how to join or where to go	-	-	12	6.8	-	-	-	-
	Don’t know of any clubs	2	1.0	-	-	-	-	5	2.3
	Progression – not enough/too much	2	1.0	1	0.6	-	-	1	0.5
	Don’t want to miss lunch	-	-	-	-	1	0.4	-	-
	Parent not available/no transport[a]	6	3.0	13	7.4	-	-	-	-
	Transport[a]	-	-	-	-	4	1.5	14	6.3

	Parent not available for getting home[a]	-	-	-	-	8	2.9	3	1.4
	Don't know when on or forget	-	-	-	-	6	2.2	-	-
	Moving school	1	0.5	-	-	-	-	-	-
TOTAL		***84***		***91***		***31***		***40***	
**Intrapersonal**	Dislike getting hot (+red/sweaty at 12y)	1	0.5	1	0.6	2	0.7	1	0.5
	Not available to both sexes	1	0.5	-	-	4	1.5	1	0.5
	Boring	-	-	3	1.7	-	-	-	-
	Quit	-	-	3	1.7	-	-	3	1.4
	Don't want to/not bothered	4	2.0	5	2.8	30	11.0	18	8.1
	Dislike sport	7	3.5	1	0.6	13	4.8	12	5.4
	Pain or injury/scared of getting hurt/too tired (+lazy at 12y)	10	5.0	5	2.8	18	6.6	15	6.8
	No good at sport or too hard (+ 'not sporty' at 12y)	12	5.9	11	6.3	15	5.5	5	2.3
	Age or stature	31	15.3	6	3.4	-	-	-	-
	None that appeal (+ boring at 12y)	1	0.5	-	-	53	19.5	24	10.8

	Dislike teacher	1	0.5	-	-	-	-	1	0.5
	Dislike strangers	-	-	-	-	2	0.7	-	-
	Shy/embarrassed/lack confidence	-	-	-	-	8	2.9	7	3.2
	Prefer to go home	-	-	-	-	5	1.8	-	-
	Prefer internet	-	-	-	-	1	0.4	1	0.5
	Can't swim	1	0.5	-	-	-	-	-	-
	Homework	-	-	-	-	7	2.6	6	2.7
TOTAL		***69***		***35***		***158***		***94***	

**Social environment**	Other people	1	0.5	3	1.7	-	-	-	-
	No places left or not picked for team	2	1.0	3	1.7	3	1.1	-	-
	Does other clubs or activities	6	3.0	11	6.3	31	11.4	17	7.7
	No time or clashes with other things	39	19.3	29	16.5	22	8.1	28	12.7
	Misbehaved	-	-	2	1.1	-	-	-	-
	Older people are there	-	-	-	-	2	0.7	-	-
	Bullied or don't fit in	-	-	-	-	3	1.1	4	1.8
	Friends don't go	-	-	-	-	8	2.9	10	4.5

	Play with friends or family	-	-	-	-	10	3.7	20	9.0
	Family commitments	1	0.5	1	0.6	3	1.1	7	3.2
	Lasts a long time/unsocial hours	-	-	1	0.6	-	-	1	0.5
	Clubs are on at the same time	-	-	-	-	-	-	-	-
	Insufficient opportunity	-	-	-	-	1	0.4	1	0.5
TOTAL		***49***		***50***		***83***		***88***	
	TOTAL ANSWERS GIVEN	***202***		***176***		***272***		***222***	
Total number of subdomains identified		***24***		***24***		***29***		***28***	
	**'No identifiable barrier to participation'**	**248**		**260**		**183**		**224**	

*** Numbers of responses given, and number of responses as % of total responses for each age and location of sports club (% may not add up to 100 due to rounding)**

[a] When coding responses, these appeared to be given as distinct answers at 12y, but combined at 9y
